# Allograft and prostatic involvement in a renal transplant recipient with disseminated tuberculosis

**DOI:** 10.4103/0971-4065.62097

**Published:** 2010

**Authors:** P. Sreejith, V. Jha, H.S. Kohli, M. Rathi, K.L. Gupta, V. Sakhuja

**Affiliations:** Department of Nephrology, Postgraduate Institute of Medical Education and Research, Chandigarh - 160 012, India

**Keywords:** Genitourinary tuberculosis, renal transplantation

## Abstract

Tuberculosis is a serious opportunistic infection in renal transplant recipients and is disseminated in nature in one-third of patients. Genito urinary tuberculosis is rare in renal transplant recipients. We report a patient presenting 5 years after renal transplantation with disseminated tuberculosis and allograft and prostatic involvement.

## Introduction

Tuberculosis is one of the most significant opportunistic infections in transplant recipients worldwide. The frequency of *Mycobacterium tuberculosis* infection in most developing countries ranges from 1.2% to 6.4% and upto 12%[1] in countries where tuberculosis is endemic, such as India. Although tuberculosis is not uncommon in renal transplant recipients in India, genitourinary involvement is rarely encountered. We report a patient with disseminated tuberculosis who developed allograft and prostatic involvement.

## Case Report

A 30-year-old renal transplant recipient presented with fever of 2-month duration in August 2008. Five years back, he had undergone renal transplantation with his sister as donor. His basic kidney disease was unknown and he was on triple immunosuppression with cyclosporine (CsA), azathioprine (AZA) and prednisolone. His baseline serum creatinine was 1.2 mg/dl. In addition to the fever, he also complained of malaise and weight loss of 5 kg during the last 2 months. He had no history of cough, expectoration, loose stools, dysuria or increased urinary frequency. He had no history of tuberculosis in the past.

On examination, he was febrile and had pallor but there were no palpable lymph nodes. His blood pressure was 130/90 mmHg. Examination of the various organ systems was essentially unremarkable.

**Figure 1:**
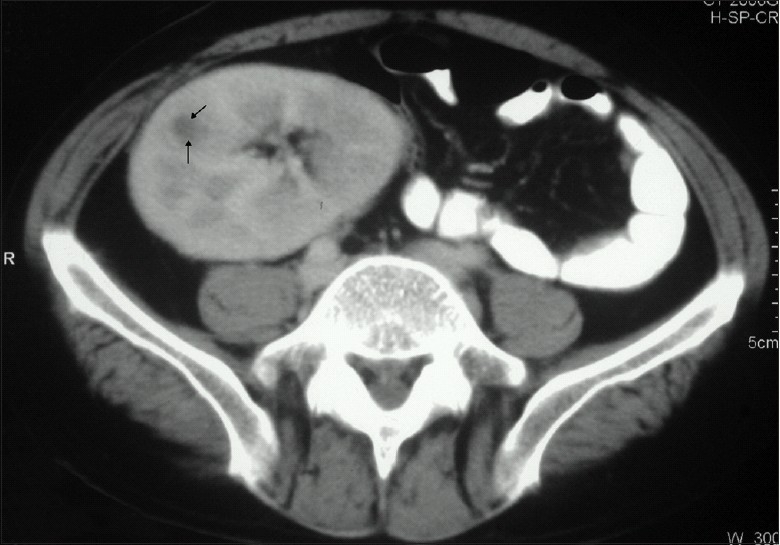
CT scan showing a hypodense lesion in the allograft

**Figure 2:**
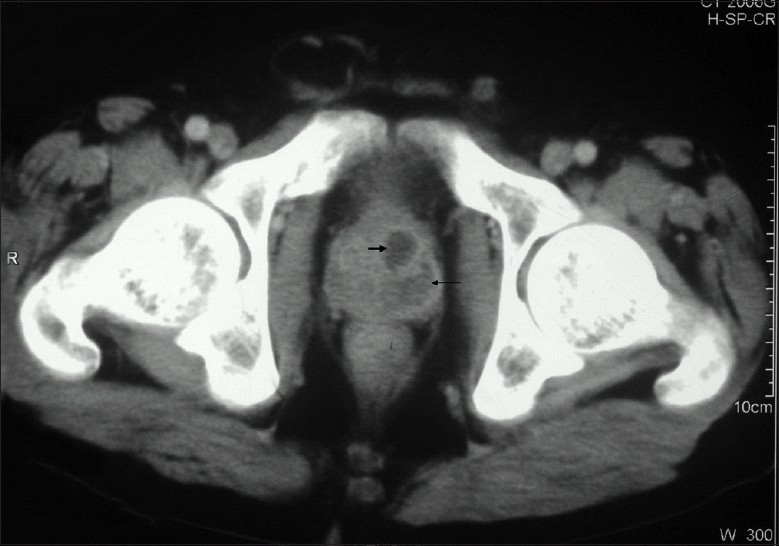
CT scan showing hypodense lesions in the prostate

His hemoglobin was 9.2 g/dl, total leukocyte count 2400/mm^3^, platelet count of 160 × 10^3^/ mm^3^, erythrocyte sedimentation rate 30 mm/1^st^ hour, serum creatinine was 1 mg/dl, sodium 142 mEq/L, potassium 4.7 mEq/L, serum bilirubin 0.73 mg/dl, aspartate aminotransferase 40 IU/L, alanine aminotransferase 26 IU/L, alkaline phosphatase 446 IU/L, serum total protein 7.5 g/dl, serum albumin 3.4 g/dl and the fasting serum glucose was 104 mg/dl. Ultrasonography of the abdomen revealed 5 × 6 cm hypo-echoic lesion in segment V of the liver. A contrast- enhanced computed tomographic scan of the thorax and abdomen revealed multiple, confluent hypodense lesions in the liver, spleen, the renal allograft [Figure 1] and prostate [Figure 2], along with multiple enlarged lymph nodes in the mesentery with central necrosis. The prostate showed multiple air specks also. The high-resolution computed tomography of lungs showed typical miliary pattern. Fine-needle aspiration cytology from the lesion in the liver showed numerous acid-fast bacilli; acid- fast bacilli were also demonstrated in the overnight urine sample. A bone marrow examination, which was performed in view of persistent leucopenia, showed hypocellularity with megaloblastosis, but there were no acid-fast bacilli or granuloma seen. HBsAg (hepatitis B surface antigen) and anti-HCV (hepatitis C virus) antibody were negative, HIV serology was non-reactive and his serum was negative for Cytomegalovirus DNA. Urine cultures performed repeatedly were sterile despite persistent pyuria.

He was started on 4-drug anti-tuberculous therapy (ATT) with isoniazid, ciprofloxacin, ethmbutol and pyrazinamide. AZA was stopped in view of the severe leucopenia. The cyclosporine dose was also reduced. He became afebrile by the end of first week of ATT. He also required treatment with granulocyte colony-stimulating factor as the leucopenia worsened further despite discontinuation of AZA.

## Discussion

Tuberculosis is a significant problem in transplant recipients in developing countries. The reported incidence from India is 11.8% to 12.3%[1,2] among renal transplant recipients. The risk of developing tuberculosis after kidney transplantation has been estimated to be 50 to 100 times higher than in the general population. The most common presentation is with pleuropulmonary involvement (51%) and disseminated tuberculosis (33%), with extra-pulmonary tuberculosis accounting for about 16%.[3] Tuberculosis in renal transplant recipients develops mainly because of the reactivation of an old, pre-existing focus.[4] The high percentage of military spread in the transplant population is generally explained by their continued treatment with immunosuppressive agents that depress T cell immunity, which is the major defense mechanism against tuberculosis. Receipt of OKT3 is a predictor of disseminated tuberculosis.[5] The optimal dose of maintenance Immunosuppression in renal transplantation is not established. Atasever *et al.* [6] reported that potent Immunosuppression with tacrolimus or mycofenolate mofetil is associated with the development of tuberculosis earlier in the post-transplant period and in younger patients when compared with conventional therapy. The maintenance use of steroid was highly associated with post-transplant tuberculosis, especially in patients maintained on a relatively high-dose steroid because of frequent acute rejection. Disseminated nature of tuberculosis in transplant recipients might be the result of delay in diagnosis due to atypical presentation or the result of excessive immunosuppression. Minimization of immunosuppression, along with administration of antitubercular drugs may be appropriate in disseminated tuberculosis after transplantation, but there are no data on the role of reduction of Immunosuppression in this group of patients.

The incidence of genitourinary tuberculosis (GUTB) among renal transplant recipients is unknown, with only about 18 cases reported in literature till date.[7–9] GUTB in a renal transplant recipient usually develops in the setting of disseminated tuberculosis. In the case series reported by Dowdy *et al*,[8] 6 out of 11 cases had features to suggest involvement at another site with tuberculosis outside the genitourinary tract. Lee *et al.*[10] has reported a case of prostatic abscess of tubercular origin in a renal transplant recipient. There are also a few case reports of tuberculous prostatic abscesses in HIV-infected patients.[11]

In the immunocompetent host, GUTB develops 8 to 39 years after primary infection. In contrast, GUTB was recognized within 6 months of transplantation in 5 out of 11 renal transplant patients reported by Dowdy *et al*.[8] Reactivation of a pre-existing renal or extra-renal focus in the face of immunosuppression is responsible for most of the cases and responsible for early presentation; however, the donor kidney may also be the source of infection.

The clinical presentation of GUTB in renal transplant recipients has been shown to be different from immunocompetent people. Genitourinary symptoms are less likely in immunosuppressed patients. While 72% of immunocompetent patients had urinary symptoms in the form of dysuria, frequency, nocturia and urgency, only 2 out of 11 renal transplant recipients with GUTB had urinary symptoms.[8] Urine mycobacterial cultures were positive in all transplant recipients with GUTB, and none of the characteristic abnormalities on intravenous pyelography seen in immunocompetent patients with GUTB were seen in transplant recipients. It has been suggested that corticosteroid therapy in renal transplant recipients may account for the lack of specificity of intravenous pyelography findings and the utility of this investigation has been questioned in this population.[8]

Our patient had involvement of the allograft and the prostate, with the contrast-enhanced computed tomographic scan showing prostatic abscess. In the setting of disseminated tuberculosis, with acid-fast bacilli being demonstrated in urine and resolution of the abscess after ATT, along with the absence of any urinary symptoms, it was presumed that the prostatic abscess was tubercular in nature.
